# Examining temporal trends in psychological distress and the co-occurrence of common substance use in a population-based sample of grade 7–12 students from 2013 to 2019

**DOI:** 10.1007/s00127-024-02619-z

**Published:** 2024-02-05

**Authors:** J. Halladay, M. Sunderland, C. Chapman, R. Repchuck, K. Georgiades, A. Boak, H. A. Hamilton, T. Slade

**Affiliations:** 1 The Matilda Centre for Research in Mental Health and Substance Use, University of Sydney, Level 6, Jane Foss Russell Building, G02, Camperdown, NSW 2006 Australia; 2 Offord Centre for Child Studies, McMaster University, Hamilton, ON Canada; 3 The Centre for Addiction and Mental Health (CAMH), University of Toronto, Toronto, Canada

**Keywords:** Psychological distress, Alcohol, Binge drinking, Cannabis, Tobacco smoking, Adolescents

## Abstract

**Purpose:**

Characterizing trends and correlates of adolescent psychological distress is important due to observed global increases over the last 20 years. Substance use is a commonly discussed correlate, though we lack an understanding about how co-occurrence of these concerns has been changing over time.

**Methods:**

Data came from repeated, representative, cross-sectional surveys of grade 7–12 students across Ontario, Canada conducted biennially from 2013 to 2019. Poisson regression with robust standard errors was used to examine changes in the joint association between psychological distress (operationalized as Kessler-6 [K6] scores ≥ 13) and substance use over time. Weighted prevalence ratios (PR) and their 99% confidence intervals were estimated, where *p* < 0.01 denotes statistical significance.

**Results:**

The prevalence of psychological distress doubled between 2013 and 2019, with adjusted increases of about 1.2 times each survey year. This biennial increase did not differ based on sex, perceived social standing, school level, or any substance use. Students using substances consistently reported a higher prevalence of psychological distress (between 1.2 times and 2.7 times higher). There were similarly no differential temporal trends based on substance use for very high distress (K6 ≥ 19) or K6 items explored individually.

**Conclusion:**

Psychological distress steeply increased among adolescents and substance use remains important to assess and address alongside distress. However, the magnitude of temporal increases appears to be similar for adolescents reporting and not reporting substance use.

**Supplementary Information:**

The online version contains supplementary material available at 10.1007/s00127-024-02619-z.

## Background

Understanding contemporary correlates of psychological distress is particularly important due to global changes in the epidemiologic landscape of mental health among adolescents over the past 20 years. There have been increases in psychological distress, depression, anxiety, and suicidality (collectively referred to as emotional concerns) among adolescents in high-income countries that began around 2009–2011, with notable spikes starting around 2012–2014 [1–6]. Specifically, the prevalence of mood and anxiety disorders increased from 9.2% in 1982 to 13.2% in 2014 among Canadian adolescents in Ontario [2]. Increases in anxiety, depression, and suicidality during similar timeframes were also observed among adolescents—more pronounced among females—in Australia, the US, and Sweden [1, 6, 7]. More recently, between 2009 and 2019, past-year major depressive episodes nearly doubled for US adolescents (8.1–15.8%), with steeper increases among females [3, 5]. Alongside these recent population-level increases, adolescent mental health-related hospital visits have increased over 100%, predominantly driven by depression, suicidality, and self-harm [8–10]. To aid in preventing further escalation, and support the reversal of these trends, understanding correlations between known risk factors and emotional concerns during these periods of fluctuation is critical.

Substance use is a commonly discussed correlate and possible risk factor for emotional concerns. Substance use is more common among adolescents with emotional concerns; for example, a study on adolescents admitted to hospital predominantly for emotional disorders reported common substance use (i.e., alcohol, cannabis, and cigarette use) at levels two-to-nine times higher than the general population [11]. Substance use has also been associated with onset and worsening of emotional concerns [12–16]. Recently, the prevalence of common substance use has been declining, plateauing, or showing only modest increases [17–21]. Despite these population-level declines in substance use, rates of emergency department visits attributable to alcohol use have increased among adolescents [22]. It is unclear why population-level trends related to emotional concerns and substance use are divergent, and we currently lack insight into how their *co-occurrence* has been changing over time [23].

Population studies—that include adolescents—examining trends in the co-occurrence of substance use and emotional concerns (largely focused on depression) have found mixed results depending on the years covered, country, sample, type of substance use, and approach to measurement [23]. In brief, there is some evidence of *weakening* of associations between emotional concerns and binge drinking [24, 25], frequent cannabis use [15], and smoking [26]. However, other studies demonstrate *strengthening* in the co-occurrence of emotional concerns and cannabis use [25, 27–29], alcohol use [30–33], and smoking [26, 30, 34]. Further, several have found *consistency* in the magnitude of positive associations over time between emotional concerns and alcohol use [15, 30, 33], cannabis use [15, 30, 35], and smoking [15, 25, 30]. Of note, only two of these studies accounted for behavioral concerns in their analyses (e.g., antisocial behaviors; [25, 30]), which has been found to partially account for associations between substance use and emotional concerns [36, 37]. The InterSECT Framework has been proposed as a way to conceptualize, evaluate, and understand changes in co-occurrence over time [23]. Strengthening (or hardening), staying the same (or consistency), or weakening (or decoupling) suggest different changes in the shared and independent risk and protective factors related to substance use and emotional concerns over time. Understanding these joint trends will thus have important implications for prevention and early intervention initiatives.

Regardless of changes over time, most studies demonstrate consistent elevations in emotional concerns among adolescents who use substances, compared to those who do not. Many aforementioned studies report stronger associations among females [24, 27–29], though not all [15]. Sex differences may be in part be due to: biological sex differences, related to hormones, genes, and physiology; and/or gender differences, related to values, beliefs, stigma, and discrimination [38]. Substance use and emotional concerns also fluctuate across development, with both increasing in prevalence as adolescents age; some studies suggest co-occurrence is stronger in early adolescence and gradually diminishes in later adolescence as both become more prevalent [14, 28, 39, 40]. These developmental differences often align with the transition from elementary (often, kindergarten to grade 8) to secondary (grades 9–12) school in Canada, which concurrently represents a socio-contextual shift related to school environment and social norms. Further, there is a concern regarding growing disparities for adolescents with low socioeconomic status (SES), with stronger relationships between higher SES and substance use in more recent years [41] and some evidence that strengthening of co-occurrence may be disproportionately larger for adolescents with lower SES [32]. There exist limited and mixed findings regarding whether trends in co-occurrence are differentially changing across sex, development, and SES for different substances.

The objectives of this study were to: (1) quantify the magnitude of change in prevalence of psychological distress among Ontario grade 7–12 students between 2013 and 2019; (2) quantify the degree of co-occurrence between psychological distress and alcohol use, cannabis use, and cigarette smoking and examine whether the strength of associations changed over time; and (3) explore whether the trends in psychological distress and co-occurring distress and substance use differed based on school level (elementary/middle vs. secondary), sex, or perceived social standing (SES-indicator).

## Methods

### Sample and data

The Ontario Student Drug Use and Health Survey (OSDUHS) is a provincially representative repeated cross-sectional survey of grade 7–12 students in publicly funded schools across Ontario,^1^ Canada conducted every 2 years. In Ontario, most young people attend publicly funded schools, and thus, out-of-scope schools, classes, and students are estimated to be only 6–9% of the total Ontario student population across survey years (See Boak [21]). Notably, in Canada, elementary schools typically include students in Junior Kindergarten up to grade 8, with few attending separate middle schools, followed by a transition to a separate secondary school that includes students in grades 9–12. Students in grades 7–8 are roughly 12–14 years of age, corresponding with early adolescence, while those in grades 9–12 are roughly 14–18 years of age, corresponding with middle and late adolescence. Ethics approval was obtained by the Research Ethics Boards at the Centre for Addiction and Mental Health (CAMH) and York University as well as all participating school board research review committees.

Sampling followed a two-stage cluster design wherein schools were first selected through probability proportionate-to-size sampling followed by the random selection of 1–2 classes per grade (See Boak [21] and Supplementary Materials [SM1] for more details). Signed parental consent and student assent were required. The paper and pencil surveys were anonymous, facilitated by research staff during regular class times, and took 30 min on average to complete. A split-ballot questionnaire was used with two main types of forms: Form A, containing extended information on mental health and Form B, containing extended information about substance use. The forms were distributed alternately (i.e., A, B, A, B) in classes to create near-equal random samples for each form. This analysis used data from Form A in 2013, 2015, 2017, and 2019. Across included years, the response rate ranged from 50 to 63% for schools, 87–94% for classes, and 59–62% for students. The final sample for analysis included 24,846 students.

### Measures

#### Psychological distress

Psychological distress was measured with the Kessler-6 (K6), a 6-item measure that asks about frequency of feeling nervous, hopeless, restless or fidgety, depressed, that everything was an effort, and worthless over the past 4 weeks [42]. Response options follow a Likert scale of 0 “none of the time” to 4 “all of the time.” Scores were summed, 0–24, with higher scores reflective of greater distress. The measure has demonstrated good psychometric properties among samples of adolescents [43, 44]. A series of Confirmatory Factor Analyses was performed in the current sample, suggesting that a single factor structure was appropriate and invariant across sex and survey year (details in SM2). A score of ≥ 13 is the most commonly used cut-off for serious psychological distress [42, 45–48], though ≥ 8 has been suggested for moderate-to-serious distress [21, 46] and ≥ 19 for very high distress [49]. Therefore, a score of ≥ 13 (serious) was used for primary analyses and sensitivity analyses were conducted with ≥ 8 (moderate-serious) and ≥ 19 (very high). In further sensitivity analyses, each individual item was explored separately (dichotomized as 1 = most or all of the time).

#### Alcohol use

Students were asked, “*In the last 4 weeks, how often did you drink alcohol (liquor, wine, beer, or coolers)?*” with 8 response options from never in their lifetime or not in the last 4 weeks to more than once each day. Regarding heavy episodic drinking (HED), students were asked, “*How many times in the last 4 weeks have you had 5 or more drinks of alcohol on the same occasion?*” with 8 response options from never in their lifetime to 5 or more times. Based on cell counts and prior temporal papers, response options were collapsed into any past month alcohol use and any past month HED (both 0 = no, 1 = yes).

#### Cannabis use

Students were asked, “*In the last 4 weeks, how often (if ever) did you use cannabis (also known as marijuana, “weed”, “pot”, “grass”, hashish, “hash”, hash oil, etc.)?*” with 8 response options from never used in lifetime to more than once each day. Response options were collapsed into any past month cannabis use (0 = no, 1 = yes).

#### Cigarette smoking

Students were asked, *“In the last 12 months, how often did you smoke cigarettes?”* with 11 response options from never in their lifetime to 30 or more cigarettes a day. Monthly smoking was not captured on Form A, and thus, response options were collapsed into any past-year cigarette smoking of more than a few puffs (0 = no, 1 = yes).

#### Covariates and moderators

Covariates and moderators include age in years, sex (1 = female, 0 = male), perceived social standing of family in society (single item related to money, education, jobs, respect captured from 1 to 10, centered at the mean of 7; [50]), race (Equity deserving racial groups, Multiracial, White = reference; groups collapsed based on cross-tabs), immigrant status (1 = youth who were foreign-born or who had at least 1 foreign-born parent; [51]), family structure (1 = living in one home with two biological or adoptive parents; [52]), school level (0 = elementary/middle, 1 = secondary), and antisocial behaviors. Antisocial behaviors were based on ≥ 3 endorsements of the following 10 behaviors ≥ 1 time in the past 12 months [21]: took a car for a ride without the owner’s permission, damaged something on purpose that did not belong to you, sold marijuana or hashish, took things worth $50 or less, took things worth more than $50, beat up or hurt anyone on purpose, broke into a locked building other than own home, carried a weapon such as gun or knife, ran away from home, and/or set something on fire you were not supposed to. Finally, the following question related to gender identity was only asked of secondary students in 2017 and 2019: “How would you describe yourself?” with response options male, female, transgender, none of the above, and prefer not to answer. For an exploratory analysis using this subset of data, these responses were combined with sex and grouped into cis-gender males, cis-gender females, and transgender or gender diverse (prefer not to answer was treated as missing).

### Analysis

Poisson regression with robust standard errors was used to examine changes in the prevalence of psychological distress and joint associations between distress and substance use over time in Stata 15.1SE. Time-varying effect models were initially run to explore non-parametric trends; best-fitting models suggested linear trends with possible inflections at grade 9, suggesting that linear models with school-level interactions were sufficient ([53]; See SM3). Poisson regression enables direct modeling of the probability or prevalence conditional on model predictors and covariates [54, 55]. To address objective one, main effects of year, substance (all substances included in separate models), school level, sex, perceived social standing, and age were assessed (Model 1). For objective two, 2-way interactions were added between year and substance (Model 2). For objective three, 3-way interactions (and lower order 2-way interactions) were added between year, substance, and school level, sex, or perceived social standing (Model 3). Fully adjusted models (Model 4) were assessed by including all significant interactions from prior models and extended socio-demographics. Prevalence ratios (PRs; i.e., risk ratios in cross-sectional data) and 99% confidence intervals were estimated, where *p* < 0.01 denotes statistical significance due to multiple testing. PRs of 1.32, 1.91, and 2.48 represent small, medium, and large population effects, respectively [56]. Models included survey year modeled continuously, recoded 0–3.^2^ All statistics accounted for the complex survey design (i.e., strata, school clustering,^3^ and sampling weights). For sensitivity analyses, Model 4 was expanded to assess all substances simultaneously, Models 1–4 were explored for moderate-severe distress (K6 ≥ 8), and Models 1–2 were explored for very high distress (K6 ≥ 19) and single items. Finally, PRs based on gender identity were explored among secondary students by pooling 2017 and 2019 survey cycles.

Prior to examining missingness, within-person mean substitution was used within the K6 score for those with up to 2 (out of 6) items missing. Of the main variables, 94.6% were complete cases. When including extended demographics, there were 87.4% complete cases. Specifically, 98.7% had complete psychological distress data and 97.8% had complete substance use responses. However, 10.8% of the sample had at least one covariate variable missing, with the highest missing on antisocial behaviors (3.8%), followed by race (3.3%), and perceived social standing (2.4%). To explore differences between students with and without missing data, a series of Poisson regressions were performed whereby any missing was coded as a 1 and those with complete data were coded as 0. Missingness was significantly more likely among students who were younger, in elementary school, male, used alcohol, reported antisocial behaviors, and who did not live with two parents in a single home. No other demographics or substances significantly predicted missingness (See SM4). Prior to main Poisson regressions, missing data were imputed through multiple imputations by chained equations (10 imputations, MICE; using the Stata command *mi impute* chained). Final models were run, accounting for the survey structure, pooling estimates across imputations, and adjusting standard errors (using the Stata commands *mi estimate: svy: poisson*). Descriptive statistics were also based on weighted and imputed data.

## Results

The unadjusted weighted prevalence of serious psychological distress increased from 10.5% in 2013 to 20.3% in 2019 (See Table 1). Across all adjusted models, the prevalence of serious psychological distress increased about 1.2 times each survey year (See Table 2). This increase did not differ based on sex, perceived social standing, school level, or any substance (See Model 2 and SM5^4^).

**Table 1:** Weighted and pooled descriptive statistics, presented as percentage or mean (standard error)

	Total	2013	2015	2017	2019	Relative difference 2013–2019
Past month psychological distress						
Moderate–serious (K6 ≥ 8)	34.5%	23.1%	33.5%	38.3%	43.3%	+ 187%
Serious (K6 ≥ 13)	15.4%	10.5%	13.9%	16.9%	20.3%	+ 193%
Very high (K6 ≥ 19)	3.5%	2.0%	3.1%	4.4%	4.5%	+ 225%
Substance use						
Past month alcohol	31.1%	34.1%	30.5%	31.6%	28.0%	− 18%
Past month heavy episodic drinking	17.6%	20.4%	17.8%	17.1%	14.8%	− 27%
Past month cannabis	13.7%	14.5%	13.7%	12.6%	14.2%	− 2%
Past year cigarette smoking	7.3%	8.4%	8.6%	7.4%	5.0%	− 40%
Socio-demographics						
Female sex	48.4%	47.8%	48.3%	48.6%	48.8%	–
Gender^a^						
Cis-gender male	50.8%	n/a	n/a	50.6%	51.0%	–
Cis-gender female	48.1%	n/a	n/a	48.1%	48.0%	–
Transgender or gender diverse	1.1%	n/a	n/a	1.3%	1.0%	–
Secondary school	75.1%	75.5%	71.7%	76.8%	76.1%	–
Perceived social standing (centered at 7)	0 (0.023)	0 (0.04)	0 (0.05)	-0.1 (0.05)	-0.01 (0.04)	–
Age	15.1 (0.03)	15.2 (0.08)	15.1 (0.06)	15.2 (0.08)	15.1 (0.05)	–
Immigrant status	51.7%	46.6%	50.6%	55.5%	54.3%	–
Two parents in one home	69.4%	71.2%	71.1%	68.7%	66.6%	–
Race						
White	57.1%	62.2%	59.6%	54.8%	51.6%	–
Equity deserving racial groups	32.6%	29.6%	30.9%	33.6%	36.4%	–
Multiracial	10.3%	8.2%	9.5%	11.5%	11.9%	–
Antisocial behaviors	11.7%	11.5%	9.2%	12.9%	13.2%	–

^a^Only included in 2017 and 2019 in secondary schools

**Table 2:** Poisson regressions predicting serious psychological distress, presented as PR (99% confidence interval); *p* value

	Alcohol		HED		Cannabis		Cigarette smoking	
	Model 2	Model 4	Model 2	Model 4	Model 2	Model 4	Model 2	Model 4
Year	1.24* (1.16–1.32); < 0.001	1.22* (1.16–1.28); < 0.001	1.21* (1.15–1.29); < 0.001	1.22* (1.16–1.28); < 0.001	1.24* (1.17–1.31); < 0.001	1.21* (1.15–1.27); < 0.001	1.25* (1.18–1.32); < 0.001	1.23* (1.17–1.29); < 0.001
Substance	1.46* (1.14–1.87); < 0.001	1.34* (1.18–1.52); < 0.001	1.24 (0.95–1.60); 0.035	1.21* (1.05–1.39); 0.001	1.75* (1.31–2.33); < 0.001	2.68* (1.71–4.19); < 0.001	2.23* (1.65–3.01); < 0.001	1.74* (1.48–2.04); < 0.001
Year*substance	0.99 (0.90–1.10); 0.865		1.05 (0.94–1.18); 0.242		0.95 (0.84–1.08); 0.313		0.94 (0.82–1.08); 0.248	
Secondary	1.08 (0.84–1.39); 0.452	1.09 (0.85–1.39); 0.363	1.11 (0.86–1.44); 0.292	1.12 (0.87–1.43); 0.246	1.1 (0.85–1.41); 0.358	1.15 (0.91–1.46); 0.133	1.12 (0.88–1.44); 0.226	1.12 (0.88–1.43); 0.207
Substance*secondary						0.5* (0.32–0.79); *p* < 0.001		
Female sex	2.72* (2.36–3.14); *p* < 0.001	2.8* (2.43–3.23); < 0.001	2.74* (2.38–3.16); *p* < 0.001	2.82* (2.45–3.24); *p* < 0.001	2.79* (2.42–3.20); *p* < 0.001	2.84* (2.47–3.26); *p* < 0.001	2.81* (2.44–3.22); *p* < 0.001	2.86* (2.49–3.28); *p* < 0.001
Perceived social standing	0.82* (0.79–0.84); *p* < 0.001	0.83* (0.80–0.86); < 0.001	0.82* (0.79–0.84); *p* < 0.001	0.83* (0.81–0.86); *p* < 0.001	0.82* (0.80–0.85); *p* < 0.001	0.84* (0.81–0.87); *p* < 0.001	0.82* (0.79–0.85); *p* < 0.001	0.84* (0.81–0.87); *p* < 0.001
Age	1.07 (1.00–1.14); 0.01	1.07* (1.003–1.14); 0.007	1.08* (1.01–1.15); 0.004	1.08* (1.01–1.15); 0.002	1.08* (1.005–1.15); 0.006	1.08* (1.01–1.15); 0.004	1.07* (1.01–1.14); 0.004	1.07* (1.01–1.14); 0.003
Immigrant status		1.04 (0.88–1.24); 0.515		1.03 (0.87–1.22); 0.632		1.04 (0.88–1.22); 0.586		1.05 (0.89–1.23); 0.476
Family structure		0.85* (0.73–0.99); 0.005		0.85* (0.73–0.98); 0.003		0.86* (0.74–0.99); 0.007		0.87 (0.75–1.004); 0.012
Equity deserving racial groups		1.03 (0.83–1.26); 0.756		1 (0.81–1.23); 0.958		1 (0.81–1.23); 0.966		1 (0.82–1.23); 0.983
Multiracial		1.18 (0.98–1.42); 0.02		1.18 (0.98–1.42); 0.02		1.17 (0.97–1.41); 0.031		1.18 (0.99–1.42); 0.017
Antisocial behavior		1.64* (1.42–1.89); < 0.001		1.68* (1.46–1.93); *p* < 0.001		1.59* (1.37–1.85); *p* < 0.001		1.56* (1.36–1.80); *p* < 0.001

*p < 0.01

Though the likelihood of distress given substance use did not change over time (PR_sub*year_ ranged 0.94–1.05, with *p* values 0.2–0.9), students using substances consistently reported a higher prevalence of psychological distress (between 1.2 times and 2.7 times higher).^5^ The rate of co-occurrence with cannabis was significantly higher for elementary compared to secondary students (PR_elem_ = 2.68 [1.71–4.19]; PR_sec_ = 1.35 [1.17–1.57]). The magnitude of the substance use PRs was small for alcohol and HED, small for cannabis use among secondary students, medium for cigarette smoking, and medium–large for cannabis use among elementary students. The effects of substance use remained after adjusting for extended socio-demographics (See Model 4) and other substances (See SM5). Further, serious psychological distress was nearly three times as likely among females compared to males. Among secondary students in 2017 and 2019, there were no significant interactions between substance use and gender, though transgender and gender diverse students were over four times more likely to experience distress than cis-gender males (PR_cis-female_ = 2.6–2.7; PR_transgender_ = 4.2–4.4).

Regarding sensitivity analyses, all substances remained independently, meaningfully related to psychological distress (though slightly attenuated) when adjusted for one another. There were also no significant changes in the prevalence of co-occurrence between any substance and very high psychological distress or with any of the individual items. However, when examining lower threshold moderate-serious distress, significant joint-temporal trends emerged for cannabis, whereby there was a significant 3-way interaction between cannabis, year, and sex with additional school-level differences for females. The co-occurrence of cannabis and moderate-serious distress increased for males from PR_2013_ = 0.88 (0.59–1.31; *p* = 0.4) to PR_2019_ = 1.33 (1.07–1.65; *p* = 0.001), becoming significantly positively related in 2019. On the other hand, the co-occurrence decreased for female students in elementary school from PR_2013_ = 2.33 (1.55–3.53; *p* < 0.001) to PR_2019_ = 1.85 (1.28–2.68; *p* < 0.001) and female students in secondary school from PR_2013_ = 1.48 (1.23–1.79; *p* < 0.001) to PR_2019_ = 1.18 (1.05–1.32; *p* < 0.001). See Table 3 and SM5.

**Table 3:** Poisson regressions predicting moderate-serious psychological distress for males and females across years (stratified model 4), presented as PR (99% confidence interval); *p* value

	2013	2015	2017	2019
Males	0.88 (0.59–1.31); 0.403	1.01 (0.78–1.31); 0.92	1.16 (0.97–1.39); 0.033	1.33* (1.07–1.65); 0.001
Females elementary	2.33* (1.55–3.53); *p* < 0.001	2.16* (1.47–3.17); *p* < 0.001	2.00* (1.39–2.89); *p* < 0.001	1.85* (1.28–2.68); *p* < 0.001
Females secondary	1.48* (1.23–1.79); *p* < 0.001	1.37* (1.21–1.55); *p* < 0.001	1.27* (1.16–1.39); *p* < 0.001	1.18* (1.05–1.32); *p* < 0.001

*p < 0.01

## Conclusions

Serious psychological distress doubled between 2013 and 2019 among grade 7–12 students across Ontario, Canada. The prevalence of serious psychological distress among students using substances was between 1.2 times higher for heavy episodic drinking among all students, and 2.7 times for cannabis use among elementary students, compared to students not using substances. However, the increase in psychological distress between 2013 and 2019 did not differ based on any substance use, sex, perceived social standing, or school level, though all these factors were consistently related to distress across time. Thus, substance use remains important to consider alongside adolescent psychological distress; however, this consistent co-occurrence suggests that, on balance, adolescent distress is increasing at similar rates for those using and not using substances. This implies that the primary drivers of increased distress are likely independent from drivers of substance use.

The doubling in psychological distress over the past decade in Ontario mirrors the doubling in depression observed among US adolescents during a similar time period [3] and builds on literature across high-income countries from the past two decades documenting increases in adolescent emotional concerns [1, 2, 4–6]. Some have speculated that improved mental health literacy and reductions in stigma have led to more disclosures of emotional concerns [57]. Measurement invariance was established across time in the current study, indicating that observed changes were not explained by changes in how adolescents potentially interpreted and responded to the distress questions. Others hypothesize that increasing distress may be due to adolescents’ increasing uncertainty about their futures—related to the climate crisis [58] and economic and career-related opportunities [59]. Climate-related research is in its infancy and prior work among US adolescents did not find factors related to the economic recession to be positively related to emotional concerns [6], though these factors may have a lagged effect. The current study similarly did not find differential trends in distress based on indicators of perceived social standing. However, nuanced measures of financial hardship (e.g., relating to deprivation) have been found to be more strongly associated with mental health difficulties than other measures of socioeconomic position [60, 61], and may relate to trends differently.

Other theories for increases in the prevalence psychological distress relate to changes in how adolescents spend their time and connect [23]. There has been a notable decline in the time adolescents spend engaging in unsupervised activities with or without peers [62]. Adolescents with low levels of social and unsupervised time tend to report worse emotional symptoms than their peers, though investigations to date do not show these shifts meaningfully explaining increasing distress [62]. There are also concerns about social media displacing in-person social interaction [6]. Societal shifts toward individualized lifestyles may be further contributing to diminished social connections and increases in adolescent loneliness over time [63], which has been associated with numerous negative mental health outcomes [64]. More broadly, shifts in lifestyle factors (i.e., substance use, sleep, exercise, screen time, and nutrition) are collectively contributing to a new field of “lifestyle psychiatry” [65]. Unfortunately, many measures of social and lifestyle factors in the existing population-surveys lack the specificity needed to understand their associations with distress (considerations related to exercise [66] and social media [67, 68]).

Substance use, which decreased or remained stable during the current study, was the focus of investigation. Relative decreases between 2013 and 2019 of 2% for cannabis, 18–27% for alcohol and heavy episodic drinking, and 40% for cigarette smoking were observed; alcohol and smoking declined significantly independent from psychological distress. Though understudied, declines in alcohol use have been partly explained by shifts in parental practices and monitoring [69], while declines in cigarette smoking have largely been attributed to the implementation of prevention programs and policies [70, 71]. Cannabis use was also declining (~ 13% relative decrease from 2013 to 2017), but declines may have reversed or halted the year after national legalization [21]. Regardless of the independent trends, adolescents who used substances were consistently more likely to experience psychological distress with co-occurrence often relating to poorer functioning and prognosis [12–16]. The magnitude of this co-occurrence remained stable over time indicating that factors related to substance use were not the core drivers of distress during this time.

Stability in co-occurring trends have also been observed among North American and European adolescents: Kahn, Wilcox [15] similarly found stability in the co-occurrence of suicidality and alcohol and smoking; Gage, Patalay [30] found stability in the co-occurrence of depression and heavy drinking, cannabis, and weekly smoking (though strengthening for *any* alcohol and smoking); Weinberger et al. [35] noted consistency between depression and smoking over time; Askari et al. [25] found consistency in co-occurring past month smoking and internalizing symptoms; and Pape et al. [33] also found consistent levels of co-occurrence between past-year cannabis use and frequent alcohol intoxication with depressive symptoms (though strengthening found for past month cannabis or any alcohol intoxication). For alcohol, findings of stability conflict with studies noting decoupling of heavy drinking and depression [24, 25] and strengthening between any drinking and depression [30, 32, 33] or wellbeing [31]. For smoking, such findings conflict with those indicating strengthening between smoking and anxiety [34] and conflicting country-specific increases or decreases between smoking and depression [26]. These discrepancies may be due to differences across countries, years of observation, ages of participants, and/or the specific type of emotional concern studied. In particular, Szatkowski, McNeill [34] observed a sample of both youth and adults up to 2011; thus, the discrepancy could be due to negligible impacts of more recent tobacco-control policies on co-occurrence, differential policy impacts for adults versus adolescents, and inequities related to tobacco-cessation being more important among adults. Regardless of mechanisms, stability of co-occurrence suggests alcohol and smoking are not driving recent increases in distress.

The largest pool of literature on co-occurring trends currently exists for cannabis, with three prior studies noting stability [15, 30, 33]. Five prior studies observed strengthening in the co-occurrence of cannabis and emotional concerns, hypothesized to be due to increasing potency in cannabis over time and/or reductions in perceived risk alongside increases in perceived therapeutic benefits [24, 25, 27–29]. Only one prior study found weakening, hypothesized to be due to greater social acceptability and reductions in stigma, and thus less risk or deviancy needed to use substances [15]. Though several studies have documented rapid increases in cannabis potency globally [72], most report levels prior to 2013 and thus less is known about recent changes and/or stabilizations in cannabis potency. It is possible potency did not change considerably between 2013 and 2019. Further, the perceived risk of trying cannabis and its perceived availability have recently remained relatively stable among Ontario students [21]. However, at lower levels of distress, there were reductions in the co-occurrence among females and increases among males over time. Given females are posited to have greater biological vulnerability for co-occurring cannabis and emotional concerns, these decreases are likely being driven by gender-related factors [38, 73]. For example, changes in public perceptions may be disproportionately impacting girls, perhaps in a lagged fashion, who have historically experienced higher rates of stigma and discrimination related to substance use [15, 38]. Overall, contemporary risk and protective factors related to cannabis use may operate differently based on sex and/or gender and at different thresholds of distress.

In summary, adolescent psychological distress was increasing at similar rates for those using and not using substances. Guided by InterSECT Framework [23], this suggests that there are contemporary risk factors for psychological distress that are largely *independent* from risk factors for substance use and/or new protective factor(s) that have shielded adolescents who use substances from being disproportionately impacted. At the same time, one of the following is maintaining consistency in co-occurrence: (a) the presence and impact of prior shared risk and protective factors remain intact (i.e., *consistency*); (b) prior shared risk factors have weakened over time but have been replaced by strengthening or additions of other risk factors or weakening of protective factors (i.e., *risk replacement*); or (c) prior shared protective factors have strengthened over time but have been offset by strengthening or additions of risk factors or weakening of other protective factors (i.e., *offsetting protective factors*). These factors can operate within the individual (e.g., patterns of use, biology), microsystem (e.g., parental monitoring), mesosystem (e.g., parent-peer interactions), exosystem (e.g., school policies), macrosystem (e.g., public perceptions), and the chronosystem (e.g., climate and economic crises) [62, 74]. Given the inconsistent findings across existing trends studies, and uncertainties regarding the mechanisms driving these changes, replication and deeper characterizations of trends in co-occurrence are needed to better understand these changes.

The current study has several limitations. First, the repeated cross-sectional design prohibits any causal inferences. Second, the period of observation was prior to the COVID-19 pandemic and only one time-point occurred following national cannabis legalization, and future trends remain uncertain. Third, given previous work suggesting initial spikes in emotional concerns occurred at or prior to 2013, there may have been initial changes in the co-occurrence of substance use and distress at the time of initial fluctuations that were not observed during the period of observation for the current study. The available observation period also restricted the ability to disentangle age-period-cohort effects. Fourth, e-cigarette and more nuanced cigarette use questions were not included in Form A, and thus could not be explored in these data. Finally, several potential mechanistic factors were not measured or explored in this study.

Overall, this work demonstrates steep increases in psychological distress among adolescents and indicates that substance use remains important to consider and address alongside distress when designing and evaluating mental health and substance related policies and prevention programs. However, on balance, changes in the risk factors driving increases in distress seem to be *equally* impacting adolescents using and not using substances. Future research is needed to understand the shift in shared and independent risk and protective factors for psychological distress and substance use among younger generations of adolescents.

### Supplementary Information

Below is the link to the electronic supplementary material.

Supplementary file1 (DOCX 53 KB)

## Data Availability

The datasets analyzed for the current study are available under a data sharing agreement by contacting osduhs@camh.ca.

## Footnotes

1 Ontario is Canada’s most populous province.

2 Given TVEMs suggested linear models with no knots, apart from changes in slopes conditional on school level, year was included continuously for parsimony. Dummy-coded (i.e., categorical) year was explored and results did not differ.

3 School and strata codes were created with unique values across cycles. Thus, it is possible that the same school was included across multiple years but coded as unique within year.

4 Unweighted school intracluster correlation coefficients (ICCs) are also available in SM5.

5 Similarly, serious psychological distress was not differentially related to trends in substance use over time (i.e., non-significant distress by year interactions), though distress was consistently positively related to use (PRs ranged from 1.2 to 1.9 depending on the substance). See SM6 for full results.
