# Supplementary material for: Examining temporal trends in psychological distress and the co-occurrence of common substance use in a population-based sample of grade 7–12 students from 2013 to 2019
Source: Soc Psychiatry Psychiatr Epidemiol. 2024 Feb 5;59(8):1367–77. doi: 10.1007/s00127-024-02619-z (PMC11291599; doi:10.1007/s00127-024-02619-z)
Supplement: Supplementary file 1 — Supplementary file1 (DOCX 53 KB) [file 127_2024_2619_MOESM1_ESM.docx]

**Examining temporal trends in psychological distress and the co-occurrence of common substance use in a population-based sample of grades 7-12 students from 2013-2019**

**Supplementary Materials**

**SM1. Sample Details**

Sampling followed a two-stage cluster design wherein schools were first selected (stratified by region and school level) through probability proportionate-to-size sampling followed by the random selection of 1-2 classes per grade with equal probability. Students were not captured by the sampling design if they were: homeschooled or institutionalized; attending private schools, schools on First Nations reserves, schools on military bases, geographically remote/inaccessible schools, or schools with very low enrolment; in Special Education, English as a Second Language, or very small classes; or unable to comprehend English or French. Out-of-scope schools, classes, and students were estimated to be 6-9% of the total Ontario student population across survey years. In Canada, elementary schools typically include Junior Kindergarten up to grade 8, with few students attending separate middle schools, followed by a transition to secondary school that includes grades 9 to 12. Signed parental consent and student assent were required. The paper and pencil surveys were anonymous, facilitated by research staff during regular class times, and took 30 minutes on average to complete. A split-ballot questionnaire was used with two main types of forms: Form A, containing extended information on mental health and Form B, containing extended information about substance use. The forms were distributed alternately (i.e., A, B, A, B) in classes to create near-equal random samples for each form. This analysis used data from Form A in 2013, 2015, 2017, and 2019. Across included years, the response rate ranged from 50% to 63% for schools, 87% to 94% for classes, and 59% to 62% for students. Non-response bias was mitigated through the selection of replacement schools or classrooms and the application of adjusted population weights. The final case weights provided for analysis are based on the combination of five components including the probability of school selection, the probability of class selection, adjustments for student-level non-responses, regional adjustments, sex-by-grade adjustments. This weighting is done to ensure final estimates are representative of all students grades 7 to 12 enrolled in publicly funded schools across Ontario. The final sample for analysis included 24,846 students, representing approximately 511,835 students per year.

**SM2. Kessler-6 Confirmatory Factor Analysis**

**Methods:** A Confirmatory Factor Analysis (CFA) was performed to explore the number of underlying factors and sex-measurement invariance for the K6. Previous psychometric evaluation of the K6 in samples of youth have found discrepant findings with respect to factor structure (1 vs. 2, with differences in if/where the effort item should be included) and concerns regarding measurement invariance across sex (Cotton et al., 2021; Ferro, 2019; Green et al., 2010; Mewton et al., 2016; Peiper et al., 2015). Mean and variance adjusted diagonally weighted least squares (WLSMV) estimation with delta parameterization, utilizing probit regression, was used for factor analyses in Mplus 7.0 (Muthén & & Muthén, 1998-2010). All models included correlated latent residuals (i.e., oblique). Tenability of CFAs (accounting for sample weights, strata, and school clustering) were evaluated by model fit indices exceeding the following thresholds: CFI>=0.95, RMSEA <=0.08, and favoured the smallest WRMR that was preferably below 1 (Schreiber et al., 2006). Further, all items had to load >=0.5 in standardized metrics with an Average Variance Extracted (AVE) of around >=0.5 to support internal validity and Cronbach alpha (*a*) of >0.7 to support internal consistency (Cheung & Wang, 2017). Measurement invariance across sex was examined including: (a) configural (pooled model fit); (b) metric (loadings); and (c) scalar (thresholds) invariance (Svetina et al., 2020). Change in CFI and RMSEA were the predominant criteria for invariance testing using suggested large-sample cut-offs of >=-0.02 and <=0.03 respectively for metric invariance and traditional cut offs of >=-0.01 and <=0.01 for scalar invariance (Rutkowski & Svetina, 2014). Reporting for the CFA models follow Jackson et al. (2009).

**Results:** Weighted CFAs were compared for models with 1-factor, 2 factors with effort loading on anxiety, 2 factors with effort loading on depression, and 2 factors without the effort item as per prior papers (Cotton et al., 2021; Mewton et al., 2016). All models surpassed *a priori* model thresholds for CFI, RMSEA, factor loadings, and convergent validity, but none yielded a WRMR<1. The model with the overall best model fit was the 2-factor model, without the effort item included (anxiety *a*=0.67; depression *a=*0.88 and 0.89 with and without effort respectively); however, all 2-factor models had discriminant validity failures. Further configural, metric, and scalar measurement invariance for sex (males and females) and year (2013, 2015, 2017, 2019) was established for the 1-factor models. To note, sex invariance for the K6 has been found in other samples of Canadian youth (Ferro, 2019).

| Table 2.1 Model fit of different factor structures | | | | | | | |
| --- | --- | --- | --- | --- | --- | --- | --- |
|  | X2 | df | RMSEA | 90% CI | CFI | TLI | WRMR |
| 1 factor | 943.752 | 9 | 0.065 | 0.061 0.068 | 0.989 | 0.982 | 3.276 |
| 2 factor (effort with anxiety) | 425.828 | 8 | 0.046 | 0.042 0.050 | 0.995 | 0.991 | 2.195 |
| 2 factor (effort with depression) | 331.862 | 8 | 0.04 | 0.037 0.044 | 0.996 | 0.993 | 1.78 |
| 2 factor (no effort) | 153.292 | 4 | 0.039 | 0.034 0.044 | 0.998 | 0.996 | 1.278 |

| Table 2.2 Convergent and Discriminant Validity | | |
| --- | --- | --- |
|  | **AVE (SQRT(AVE))** | **Correlation** |
| Anxiety with effort | 0.53 (0.73) | 0.907 |
| Depression without effort | 0.82 (0.90) |  |
| Anxiety without effort | 0.56 (0.75) | 0.864 |
| Depression with effort | 0.75 (0.86) |  |
| Anxiety (no effort) | 0.56 (0.75) | 0.849 |
| Depression (no effort) | 0.82 (0.90) |  |

| Table 2.3 Measurement Invariance | | | | | | | | | | | | | |
| --- | --- | --- | --- | --- | --- | --- | --- | --- | --- | --- | --- | --- | --- |
|  | X2 | df | CFI | RMSEA | 90% CI | WRMR | Model Comparison | **Δ**X2 | df | p | **Δ**CFI | **Δ**RMSEA | Decision |
| 1 Factor Model: **Sex-invariance** (male=10949; female=13743) | | | | | | | | | | | | | |
| M1: Configural | 1016.305 | 18 | 0.988 | 0.067 | 0.064-0.071 | 3.687 | none |  |  |  |  |  |  |
| M2: Metric | 1033.484 | 23 | 0.988 | 0.06 | 0.057-0.063 | 3.828 | M1 | 71.815 | 5 | 0 | 0 | -0.007 | passed |
| M3: Scalar | 835.427 | 40 | 0.991 | 0.04 | 0.038-0.043 | 4.068 | M2 | 87.394 | 17 | 0 | 0.003 | -0.02 | Passed |
| 1 Factor Model: **Year-invariance** (2013=5449; 2015=5382; 2017=6324; 2019=7537) | | | | | | | | | | | | | |
| M1: Configural | 1032.308 | 36 | 0.991 | 0.067 | 0.063-0.071 | 3.685 | none |  |  |  |  |  |  |
| M2: Metric | 999.19 | 51 | 0.991 | 0.055 | 0.052-0.058 | 3.88 | M1 | 95.578 | 15 | 0 | 0 | -0.012 | passed |
| M3: Scalar | 857.855 | 102 | 0.993 | 0.035 | 0.033-0.037 | 4.322 | M2 | 173.94 | 51 | 0 | 0.002 | -0.02 | passed |

**SM3. Time-Varying Effect Modeling Analysis**

To examine changes in the joint association between psychological distress and substance use over time, first, a series of logistic time-varying effect models (TVEMs) were explored in SAS Enterprise Guide V7.1. TVEMs are novel flexible non-parametric regression-based approaches developed to analyze complicated trend data, made up splines (piecewise polynomials) joined at inflection points called knots (Lanza et al., 2016). TVEMs used complete cases only (unweighted n=23,676). TVEMS were estimated as a function of historical time (i.e., year) and developmental time (i.e., grade). However, the best fitting models suggested linear polynomial terms (i.e., 1) and had either 0 or 1 knot (at grade 9). Thus, TVEMs were unnecessary.

Specifically, the best fitting year-varying models were:

- DV=K6>=13, IVs=intercept only: 0 knot with a linear polynomial (i.e., 1); linear increase
- DV=K6>=13, IVs=intercept + alcohol: 0,0 knots with a linear polynomial (i.e., 1); linear effects over time (very flat ~OR=1.76)
- DV=K6>=13, IVs=intercept + HED: 0,0 knots with a linear polynomial (i.e., 1); linear effects increasing over time (~OR=1.49 to 1.85)
- DV=K6>=13, IVs=intercept + cannabis: 0,0 knots with a linear polynomial (i.e., 1); linear effects over time (consistently ~OR=2-2.2)
- DV=K6>=13, IVs=intercept + cigarettes: 0,0 knots with a linear polynomial (i.e., 1); linear trend slightly decreasing over time (~OR=3 to 2.8)

Specifically, the best fitting grade-varying models were:

- DV=K6>=13, IVs=intercept only: 1 knot with a linear polynomial (i.e., 1); inflection at grade 9
- DV=K6>=13, IVs=intercept + year: 1,1 knots with a linear polynomial (i.e., 1); inflection at grade 9
- DV=K6>=13, IVs=intercept + year + alcohol + alcohol*year: 0,0,0,0 knots with a linear polynomial
- DV=K6>=13, IVs=intercept + year + HED + HED*year: 0,0,0,0 knots with a linear polynomial
- DV=K6>=13, IVs=intercept + year + cannabis + cannabis*year: 0,0,0,0 knots with a linear polynomial (i.e., 1)
- DV=K6>=13, IVs=intercept + year + cigarettes + cigarettes*year: 1,1,1,0 knots with a linear polynomial (i.e., 1); inflection at grade 9

**SM4. Extended Missing Data Information**

|  | Unweighted | | Weighted | |
| --- | --- | --- | --- | --- |
|  | % missing | Any Missing; PR (95% CI); p-value | % missing | Any Missing; PR (95% CI); p-value |
| K6 | 1.2% | **0.77 (0.69-0.86); 0** | 1.3% | 0.85 (0.72-1); 0.053 |
| Year | 0% | 0.98 (0.94-1.02); 0.301 | 0% | 1.03 (0.97-1.1); 0.342 |
| Alcohol | 1.2% | **0.94 (0.58-0.71); 0** | 1.2% | **0.71 (0.59-0.85); 0** |
| HED | 0.5% | **0.76 (0.67-0.86); 0** | 0.4% | 0.85 (0.68-1.06); 0.156 |
| Cannabis | 0.8% | **0.72 (0.63-0.82); 0** | 0.6% | 0.93 (0.76-1.13); 0.476 |
| Cigarette Smoking | 0.3% | 0.90 (0.77-1.05); 0.174 | 0.3% | 1 (0.79-1.27); 0.982 |
| Secondary | 0% | **0.48 (0.43-0.53); 0** | 0% | **0.54 (0.45-0.64); 0** |
| Sex | 0% | **0.73 (0.69-0.78); 0** | 0% | **0.73 (0.66-0.81); 0** |
| Perceived Social Standing | 1.9% | 1.02 (0.99-1.04); 0.160 | 2.4% | 1 (0.96-1.04); 0.996 |
| Age | 0.02% | **0.80 (0.78-0.83); 0** | 0.04% | **0.85 (0.81-0.89); 0** |
| Immigrant | 1.3% | 0.93 (0.86-1.01); 0.102 | 1.3% | 1 (0.88-1.13); 0.947 |
| Family Structure | 1.5% | **0.81 (0.75-0.87); 0** | 1.6% | **0.79 (0.69-0.89); 0** |
| Equity deserving racial groups | 4.1% | 1.03 (0.93-1.15); 0.539 | 3.3% | 1.04 (0.9-1.21); 0.612 |
| Multiracial |  | 0.93 (0.81-1.07); 0.339 |  | 1.19 (0.99-1.44); 0.069 |
| Antisocial Behaviours | 3.4% | **4.05 (3.7-4.4); 0** | 3.8% | **4.15 (3.48-4.95); 0** |
| Main Missing (Model 1-3) | 96.8% |  | 94.7% |  |
| Any Missing (Model 4) | 87.5% |  | 87.3% |  |

**SM5. Extended Regression Results**

Table 5.1 School ICCs from unadjusted, unweighted multilevel logistic models.

|  | ICCs presented as percentages (%)  Mean (min, max) across imputations |
| --- | --- |
| Psychological Distress (K6 >=13) | 4.95% (4.88 to 5.05) |
| Alcohol | 26.59% (26.52 to 26.74) |
| HED | 33.31% (33.19 to 33.49) |
| Cannabis | 26.26% (26.04 to 26.58) |
| Cigarette Smoking | 24.70% (24.52 to 24.86) |

Table 5.2 Fully Adjusted Model for Serious Psychological Distress, PR (99% CI)

| K6>=13 | **Fully Adjusted Model + Other Substance**  (dropped nonsignificant interactions) |
| --- | --- |
| **Year** | 1.23 (1.17-1.29); 0 |
| **Alcohol** | 1.20 (1.05-1.38); 0.001 |
| Cigarette Smoking | 1.54 (1.28-1.85); 0 |
| Secondary | 1.14 (0.91-1.44); 0.139 |
| **Cannabis** | 2.13 (1.38-3.29); 0 |
| **Cannabis*Secondary** | 0.51 (0.33-0.79); 0 |
| Sex | 2.85 (2.48-3.27); 0 |
| Perceived Social Standing | 0.84 (0.81-0.87); 0 |
| Age | 1.06 (0.99-1.13); 0.027 |
| Immigrant Status | 1.06 (0.90-1.25); 0.348 |
| Equity deserving racial groups | 1.04 (0.84-1.27); 0.661 |
| Multiracial | 1.17 (0.98-1.41); 0.024 |
| Family Structure | 0.87 (0.76-1.01); 0.01 |
| Antisocial Behaviours | 1.48 (1.28-1.72); 0 |

Table 5.3 Sensitivity Analysis Model 2 for Very High Psychological Distress, presented as Prevalence Ratio (99% Confidence Interval); p-value

| K6>=19 | **Alcohol Model 2** | **HED Model 2** | **Cannabis Model 2** | **Cigarettes Model 2** |
| --- | --- | --- | --- | --- |
| **Year** | 1.23 (1.05-1.43); 0.001 | 1.21 (1.05-1.38); 0 | 1.25 (1.09-1.44); 0 | 1.29 (1.13-1.46); 0 |
| **Substance** | 1.17 (0.68-2.02); 0.453 | 1.14 (0.64-2.03); 0.561 | 1.98 (1.07-3.67); 0.004 | 2.75 (1.51-5.01); 0 |
| **Year*Substance** | 1.11 (0.88-1.40); 0.258 | 1.25 (0.98-1.58); 0.016 | 1.05 (0.81-1.36); 0.623 | 1.09 (0.84-1.41); 0.394 |
| Secondary | 1.05 (0.60-1.82); 0.823 | 1.07 (0.61-1.86); 0.764 | 1.04 (0.59-1.82); 0.858 | 1.09 (0.64-1.86); 0.687 |
| Sex | 3.32 (2.31-4.77); 0 | 3.36 (2.35-4.79); 0 | 3.45 (2.45-4.87); 0 | 3.52 (2.52-4.92); 0 |
| Perceived Social Standing | 0.72 (0.66-0.79); 0 | 0.72 (0.66-0.79); 0 | 0.73 (0.67-0.80); 0 | 0.73 (0.67-0.80); 0 |
| Age | 1.1 (0.98-1.23); 0.041 | 1.09 (0.97-1.22); 0.056 | 1.08 (0.97-1.21); 0.067 | 1.08 (0.97-1.19); 0.066 |

Table 5.4 Sensitivity Analysis Model 2A for Individual Items, presented as Prevalence Ratio (99% Confidence Interval); p-value

| K6 items |  | **Alcohol** | **HED** | **Cannabis** | **Cigarettes** |
| --- | --- | --- | --- | --- | --- |
| **Nervous** | **Model 1: Substance** | 1.22 (1.09-1.36); 0 | 1.09 (0.93-1.28); 0.145 | 1.24 (1.02-1.53); 0 | 1.46 (1.22-1.75); 0 |
|  | **Model 2: Year*Substance** | 0.98 (0.89-1.09); 0.668 | 1.04 (0.91-1.18); 0.448 | 0.97 (0.87-1.08); 0.447 | 1.01 (0.86-1.18); 0.899 |
| **Hopeless** | **Model 1: Substance** | 1.4 (1.21-1.63); 0 | 1.37 (1.15-1.64); 0 | 1.37 (1.10-1.70); 0 | 1.9 (1.53-2.37); 0 |
|  | **Model 2: Year*Substance** | 0.96 (0.86-1.08); 0.417 | 1.03 (0.90-1.18); 0.568 | 0.92 (0.77-1.11); 0.254 | 1.03 (0.88-1.20); 0.662 |
| **Restless or Fidgety** | **Model 1: Substance** | 1.31 (1.13-1.52); 0 | 1.26 (1.08-1.48); 0 | 1.51 (1.30-1.75); 0 | 1.76 (1.47-2.10); 0 |
|  | **Model 2: Year*Substance** | 0.95 (0.86-1.05); 0.202 | 0.99 (0.87-1.12); 0.812 | 0.91 (0.80-1.03); 0.043 | 0.96 (0.82-1.12); 0.479 |
| **Depressed** | **Model 1: Substance** | 1.46 (1.19-1.79); 0 | 1.6 (1.32-1.93); 0 | 1.72 (1.43-2.08); 0 | 2.19 (1.82-2.64); 0 |
|  | **Model 2: Year*Substance** | 1.001 (0.87-1.15); 0.979 | 1.02 (0.87-1.20); 0.784 | 0.97 (0.83-1.12); 0.553 | 1.03 (0.87-1.21); 0.68 |
| **Effort** | **Model 1: Substance** | 1.08 (0.94-1.24); 0.139 | 1.24 (1.06-1.45); 0 | 1.34 (1.17-1.53); 0 | 1.71 (1.46-1.99); 0 |
|  | **Model 2: Year*Substance** | 1 (0.89-1.11); 0.911 | 0.97 (0.85-1.11); 0.614 | 1.01 (0.90-1.13); 0.766 | 1.04 (0.92-1.18); 0.412 |
| **Worthless** | **Model 1: Substance** | 1.38 (1.18-1.61); 0 | 1.46 (1.24-1.72); 0 | 1.7 (1.45-1.98); 0 | 2.1 (1.78-2.48); 0 |
|  | **Model 2: Year*Substance** | 0.92 (0.81-1.04); 0.067 | 0.96 (0.84-1.10); 0.452 | 0.94 (0.83-1.07); 0.231 | 1.03 (0.89-1.18); 0.619 |

| Table 5.5 Sensitivity Analyses for Moderate-Severe Psychological Distress, presented as Prevalence Ratio (99% Confidence Interval); p-value | | | | | | | | |
| --- | --- | --- | --- | --- | --- | --- | --- | --- |
| **K6>=8** | **Alcohol** | | **HED** | | **Cannabis** | | **Cigarette** | |
|  | **Model 2** | **Model 6** | **Model 2** | **Model 6** | **Model 2** | **Model 6** | **Model 2** | **Model 6** |
| **Year** | 1.22 (1.16-1.27); 0 | 1.23 (1.16-1.31); 0 | 1.20 (1.15-1.25); 0 | 1.23 (1.15-1.31); 0 | 1.2 (1.16-1.25); 0 | 1.19 (1.11-1.28); 0 | 1.21 (1.17-1.26); 0 | 1.23 (1.16-1.31); 0 |
| **Substance** | 1.24 (1.05-1.47); 0.001 | 1.56 (1.23-1.98); 0 | 1.11 (0.92-1.33); 0.17 | 1.09 (0.99-1.20); 0.022 | 1.29 (1.06-1.56); 0.001 | 1.21 (0.75-1.98); 0.302 | 1.5 (1.19-1.88); 0 | 1.39 (1.22-1.59); 0 |
| **Year*Substance** | 0.98 (0.91-1.05); 0.488 |  | 1.02 (0.94-1.10); 0.612 |  | 1 (0.92-1.08); 0.968 | 1.15 (0.98-1.34); 0.026 | 0.97 (0.9-1.05); 0.519 |  |
| Secondary | 1.06 (0.93-1.20); 0.267 | 1.11 (0.98-1.26); 0.035 | 1.07 (0.95-1.22); 0.153 | 1.08 (0.96-1.22); 0.096 | 1.07 (0.94-1.21); 0.188 | 1.08 (0.96-1.23); 0.101 | 1.08 (0.95-1.22); 0.115 | 1.08 (0.96-1.23); 0.087 |
| Substance*Secondary |  | 0.74 (0.58-0.95); 0.002 |  |  |  | 0.73 (0.511.04); 0.02 |  |  |
| Sex | 1.95 (1.80-2.10); 0 | 2.15 (1.86-2.47); 0 | 1.95 (1.81-2.11); 0 | 2.15 (1.87-2.49); 0 | 1.97 (1.82-2.12); 0 | 1.96 (1.66-2.32); 0 | 1.97 (1.83-2.13); 0 | 2.17 (1.88-2.51); 0 |
| Year*Sex |  | 0.95 (0.90-1.01); 0.038 |  | 0.95 (0.90-1.01); 0.04 |  | 0.99 (0.92-1.07); 0.767 |  | 0.95 (0.89-1.01); 0.033 |
| Substance*Sex |  |  |  |  |  | 1.67 (1.12-2.50); 0.001 |  |  |
| Year*Substances*Sex |  |  |  |  |  | 0.81 (0.68-0.95); 0.001 |  |  |
| Perceived Social Standing | 0.88 (0.87-0.90); 0 | 0.9 (0.88-0.92); 0 | 0.88 (0.87-0.90); 0 | 0.9 (0.88-0.92); 0 | 0.89 (0.87-0.91); 0 | 0.89 (0.86-0.91); 0 | 0.89 (0.87-0.90); 0 | 0.89 (0.87-0.91); 0 |
| Substance*Social Status |  |  |  |  |  | 1.07 (1.02-1.13); 0.001 |  | 1.08 (1.02-1.14); 0.001 |
| Age | 1.07 (1.04-1.10); 0 | 1.07 (1.04-1.10); 0 | 1.08 (1.05-1.11); 0 | 1.08 (1.05-1.11); 0 | 1.08 (1.04-1.14); 0 | 1.07 (1.04-1.14); 0 | 1.08 (1.05-1.11); 0 | 1.07 (1.04-1.10); 0 |
| Immigrant Status |  | 1.15 (1.04-1.27); 0 |  | 1.15 (1.04-1.26); 0 |  | 1.14 (1.04-1.6); 0 |  | 1.15 (1.04-1.27); 0 |
| Family Structure (1=2 parents, 1 home) |  | 0.85 (0.79-0.92); 0 |  | 0.85 (0.79-0.92); 0 |  | 0.86 (0.80-0.93); 0 |  | 0.86 (0.79-0.93); 0 |
| Equity deserving racial groups |  | 1.05 (0.93-1.18); 0.311 |  | 1.03 (0.91-1.16); 0.568 |  | 1.03 (0.92-1.17); 0.484 |  | 1.03 (0.91-1.16); 0.534 |
| Multiracial |  | 1.13 (1.004-1.26); 0.008 |  | 1.13 (1.01-1.26); 0.007 |  | 1.13 (1.01-1.27); 0.007 |  | 1.13 (1.01-1.27); 0.006 |
| Antisocial Behaviour |  | 1.35 (1.22-1.50); 0 |  | 1.38 (1.25-1.53); 0 |  | 1.35 (1.22-1.50); 0 |  | 1.34 (1.21-1.49); 0 |

Table 5.6 Gender exploration among secondary students in 2017 and 2019, presented as adjusted Prevalence Ratio (99% Confidence Interval); p-value. Adjusted for year, substance, perceived social status, age, immigrant status, race, family structure, and antisocial behaviours.

|  | **Alcohol** | **HED** | **Cannabis** | **Cigarettes** |
| --- | --- | --- | --- | --- |
| **Cis-Female (ref=Cis-Male)** | 2.64 (2.14-3.26); 0 | 2.66 (2.16-3.27); 0 | 2.69 (2.18-3.31); 0 | 2.7 (2.20-3.31); 0 |
| **Transgender and Gender Diverse (ref=Cis-Male)** | 4.2 (2.72-6.48); 0 | 4.26 (2.80-6.49); 0 | 4.37 (2.89-6.61); 0 | 4.37 (2.96-6.47); 0 |

**SM6. Substance Use Regression Results**

Table 6.1 Predicting substance use, adjusting for extended socio-demographics and psychological distress, presented as adjusted Prevalence Ratio (99% Confidence Interval); p-value.

|  | **Outcome: Alcohol** | | **Outcome: HED** | | **Outcome: Cannabis** | | **Outcome: Cigarette Smoking** | |
| --- | --- | --- | --- | --- | --- | --- | --- | --- |
|  | **Without Interaction** | **With Interaction** | **Without Interaction** | **With Interaction** | **Without Interaction** | **With Interaction** | **Without Interaction** | **With Interaction** |
| **K6>=13** | 1.24 (1.14-1.34); 0 | 1.25 (1.09-1.44); 0 | 1.21 (1.06-1.38); 0 | 1.12 (0.91-1.39); 0.149 | 1.39 (1.20-1.62); 0 | 1.42 (1.09-1.84); 0.001 | 1.87 (1.53-2.30); 0 | 2.07 (1.49-2.86); 0 |
| **Year** | 0.95 (0.92-0.99); 0.002 | 0.96 (0.92-0.998); 0.007 | 0.92 (0.87-0.97); 0 | 0.91 (0.85-0.96); 0 | 0.97 (0.91-1.03); 0.221 | 0.97 (0.91-1.04); 0.316 | 0.84 (0.76-0.92); 0 | 0.85 (0.77-0.95); 0 |
| **Year*K6** |  | 0.99 (0.93-1.06); 0.717 |  | 1.05 (0.94-1.16); 0.262 |  | 0.99 (0.88-1.11); 0.802 |  | 0.93 (0.80-1.09); 0.257 |
| Secondary | 2.41 (1.93-3.02); 0 | 2.41 (1.93-3.02); 0 | 3.51 (2.11-5.83); 0 | 3.51 (2.11-5.83); 0 | 4.13 (1.68-10.11); 0 | 4.13 (1.68-10.11); 0 | 2.06 (0.87-4.87); 0.03 | 2.06 (0.87-4.86); 0.03 |
| Sex | 1 (0.93-1.06); 0.869 | 1 (0.93-1.06); 0.871 | 0.91 (0.81-1.02); 0.037 | 0.91 (0.81-1.02); 0.036 | 0.8 (0.70-0.91); 0 | 0.8 (0.70-0.91); 0 | 0.71 (0.57-0.88); 0 | 0.71 (0.57-0.88); 0 |
| Perceived Social Standing | 1.03 (1.01-1.05); 0 | 1.03 (1.01-1.05); 0 | 1.04 (0.99-1.08); 0.046 | 1.04 (0.99-1.09); 0.045 | 0.99 (0.94-1.04); 0.524 | 0.99 (0.94-1.04); 0.524 | 0.99 (0.93-1.06); 0.765 | 0.99 (0.93-1.06); 0.766 |
| Age | 1.25 (1.20-1.29); 0 | 1.25 (1.20-1.29); 0 | 1.38 (1.31-1.46); 0 | 1.38 (1.31-1.46); 0 | 1.3 (1.23-1.37); 0 | 1.3 (1.23-1.37); 0 | 1.41 (1.30-1.52); 0 | 1.41 (1.30-1.53); 0 |
| Immigrant Status | 0.84 (0.75-0.93); 0 | 0.84 (0.75-0.93); 0 | 0.79 (0.67-0.92); 0 | 0.79 (0.67-0.92); 0 | 0.92 (0.76-1.10); 0.218 | 0.92 (0.76-1.10); 0.218 | 0.85 (0.66-1.10); 0.111 | 0.85 (0.66-1.10); 0.11 |
| Equity deserving racial groups | 0.51 (0.45-0.59); 0 | 0.51 (0.45-0.59); 0 | 0.49 (0.40-0.60); 0 | 0.49 (0.40-0.60); 0 | 0.57 (0.44-0.74); 0 | 0.57 (0.44-0.74); 0 | 0.57 (0.43-0.77); 0 | 0.57 (0.43-0.77); 0 |
| Multiracial | 0.93 (0.82-1.06); 0.15 | 0.93 (0.82-1.06); 0.15 | 0.88 (0.70-1.10); 0.14 | 0.88 (0.70-1.10); 0.138 | 0.97 (0.77-1.22); 0.723 | 0.97 (0.77-1.22); 0.725 | 0.86 (0.63-1.17); 0.196 | 0.86 (0.63-1.17); 0.203 |
| Antisocial Behaviour | 1.7 (1.55-1.86); 0 | 1.7 (1.55-1.86); 0 | 2.1 (1.80-2.44); 0 | 2.1 (1.80-2.44); 0 | 2.81 (2.40-3.29); 0 | 2.81 (2.40-3.29); 0 | 3.39 (2.72-4.22); 0 | 3.39 (2.71-4.23); 0 |
| Family Structure (1=2 parents, 1 home) | 0.92 (0.84-0.99); 0.006 | 0.92 (0.84-0.99); 0.006 | 0.84 (0.75-0.94); 0 | 0.84 (0.75-0.94); 0 | 0.73 (0.61-0.87); 0 | 0.73 (0.61-0.87); 0 | 0.67 (0.53-0.85); 0 | 0.67 (0.53-0.85); 0 |
